# Cannabis smoking and abdominal wall reconstruction outcomes: a propensity score-matched analysis

**DOI:** 10.1007/s10029-024-02976-3

**Published:** 2024-02-22

**Authors:** S. Maskal, J. M. Foreman, R. C. Ellis, S. Phillips, N. Messer, M. Melland-Smith, L. R. A. Beffa, C. C. Petro, A. S. Prabhu, M. J. Rosen, B. T. Miller

**Affiliations:** 1 General Surgery, Cleveland Clinic, 2049 E 100th St, Desk A-100, Cleveland, OH 44106 USA; 2 Department of Biostatistics, Vanderbilt University Medical Center, Nashville, TN USA

**Keywords:** Cannabis, Incisional hernia, Abdominal wall reconstruction, Wound complications

## Abstract

**Purpose:**

Despite increasing use of cannabis, literature on perioperative effects is lagging. We compared active cannabis-smokers versus non-smokers and postoperative wound morbidity and reoperations following open abdominal wall reconstruction (AWR).

**Methods:**

Patients who underwent open, clean, AWR with transversus abdominis release and retromuscular synthetic mesh placement at our institution between January 2014 and May 2022 were identified using the Abdominal Core Health Quality Collaborative database. Active cannabis-smokers were 1:3 propensity matched to non-smokers based on demographics and comorbidities. Wound complications, 30 day morbidity, pain (PROMIS 3a-Pain Intensity), and hernia-specific quality of life (HerQles) were compared.

**Results:**

Seventy-two cannabis-smokers were matched to 216 non-smokers. SSO (18% vs 17% *p* = 0.86), SSI (11.1% vs 9.3%, *p* = 0.65), SSOPI (12% vs 12%, *p* = 0.92), and all postoperative complications (46% vs 43%, *p* = 0.63) were similar between cannabis-smokers and non-smokers. Reoperations were more common in the cannabis-smoker group (8.3% vs 2.8%, *p* = 0.041), driven by major wound complications (6.9% vs 3.2%, *p* = 0.004). No mesh excisions occurred. HerQles scores were similar at baseline (22 [11, 41] vs 35 [14, 55], *p* = 0.06), and were worse for cannabis-smokers compared to non-smokers at 30 days (30 [12, 50] vs 38 [20, 67], *p* = 0.032), but not significantly different at 1 year postoperatively (72 [53, 90] vs 78 [57, 92], *p* = 0.39). Pain scores were worse for cannabis-smokers compared to non-smokers at 30 days postoperatively (52 [46, 58] vs 49 [44, 54], *p* = 0.01), but there were no differences at 6 months or 1 year postoperatively (*p* > 0.05 for all).

**Conclusion:**

Cannabis smokers will likely experience similar complication rates after clean, open AWR, but should be counseled that despite similar wound complication rates, the severity of their wound complications may be greater than non-smokers.

## Introduction

Cannabis use has been increasing in the United States, with an estimated 35.5 million individuals aged 26 or older reportedly using cannabis as of 2020, but its impact on surgical outcomes is still under investigation [1]. Cannabis has become more accessible with policy changes, with 23 states and the District of Columbia that have legalized marijuana for recreational use as of 2023 [2]. Cannabinoid receptors are present in many tissues throughout the body and have been shown in animal models to play a role in inflammatory response [3], intestinal motility [4], and gastric secretion [5], but whether or not there are deleterious consequences in surgical patients is not well known [6].

In the setting of growing accessibility and awareness of cannabis use, it is essential for surgeons to investigate the effects of cannabis on perioperative outcomes to determine whether it needs to be considered during surgical planning and informed consent. Open abdominal wall reconstruction is a major operation with potential for serious postoperative wound and medical complications that may be perpetuated by cannabis use. Given the paucity of data surrounding the effect of cannabis use in this setting, we aim to investigate if there is an association between cannabis use and postoperative wound morbidity or reoperations in patients undergoing clean abdominal wall reconstruction with mesh.

## Methods

This retrospective review was conducted after obtaining approval under exempt status by our Institutional Review Board. Patients who underwent open, ventral hernia repair with transversus abdominis release and retromuscular synthetic mesh placement with CDC class I wounds at our institution between January 2014 and May 2022 were identified using the Abdominal Core Health Quality Collaborative (ACHQC) database. The ACHQC is a hernia-specific, prospectively collected, national database of voluntary surgeon-entered data, including patient characteristics, operative details, perioperative and long-term outcomes, as well as patient-reported outcomes [7]. Additionally, retrospective chart review was used to identify patients meeting inclusion criteria as current cannabis-smokers and non-smokers. Keywords utilized to query the electronic medical record included: “marijuana”, “THC”, “cannabis”, “cannabinoid”, “weed”, “pot”, as well as the information input in the drug use section of the EMR (“drug use: yes/no”, “drug use frequency”, and any comments entered in the drug use section). Cannabis smokers were defined as reporting regular inhaled use at their preoperative visit. The ACHQC database was then queried for analysis based on our exposed and control categories.

The primary outcome of interest was 30 day wound morbidity which is defined as surgical site occurrences (SSO), surgical site infections (SSI), and surgical site occurrences requiring procedural intervention (SSOPI) [8]. SSO encompasses wound cellulitis, seroma, wound dehiscence, or formation of an enterocutaneous fistula, and SSOPI indicates that a procedural intervention occurred. SSI includes any surgical infection [8]. Secondary outcomes of interest included 30 day all morbidity, readmissions, and reoperations. Patient-reported outcomes included pain as measured by NIH Patient-Reported Outcome Measurement Information System (PROMIS)-3a-Pain Intensity score and abdominal wall-specific quality of life as measured by the Hernia-Related Quality-of-Life (HerQLes) survey, both of which were available at baseline, 30 days, 6 months, and 1 year postoperatively. HerQLes is a hernia-specific quality-of-life survey, which is a validated score from 0 to 100, with increasing scores indicating better quality of life and a defined minimal clinically important difference of 15.6 points [9, 10]. Patient-Reported Outcomes Measurement Information System (PROMIS) 3a-Pain Intensity survey is a three question survey addressing pain over the preceding week, reported as converted T-scores of 30.7–71.8, with lower scores indicating less pain [11, 12]. The HerQLes and PROMIS surveys are routinely collected at baseline, 30 days, 6 months, and 1 year. Responses to the HerQLes and PROMIS surveys were compared using Wilcoxon rank sum tests. The Distressed Community Index (DCI) was used to compare the association of cannabis use with socioeconomic distress. The DCI is a score indexed to zip codes that was developed from US census data to describe the socioeconomic distress of a community based on seven indicators of prosperity: prevalence of high school diplomas, housing vacancies, poverty, unemployment, changes in employment, and business establishments, and the ratio of median income [13]. DCI scores range from 0, indicating no distress, to 100, indicating high distress.

Baseline characteristics were analyzed using simple descriptive and univariate statistics. Current cannabis-smokers were matched 1:3 to a group of non-smokers based on propensity scores. We estimated the propensity score with a logistic regression and included the following in the model: age, race, insurance status, body mass index (BMI), current tobacco smoker status, diabetes mellitus, chronic obstructive pulmonary disease (COPD), immunosuppressants use, inflammatory bowel disease (IBD), hepatic dysfunction, recurrent hernia, hernia width, and Ventral Hernia Working Group grade (VHWG). As retrospective, descriptive study, missing data were anticipated and were not addressed in the statistical analysis.

## Results

**Fig. 1:**
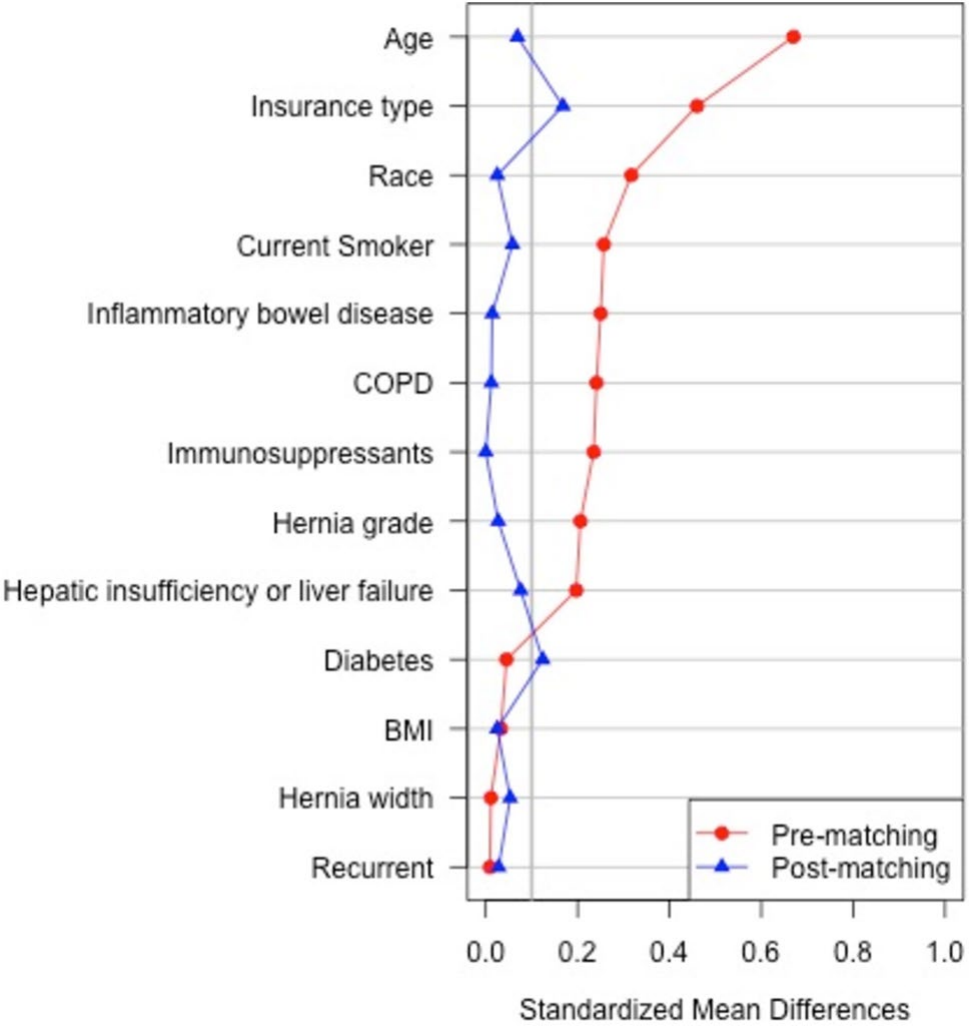
Cohort similarities before and after matching

We identified 1311 patients meeting inclusion criteria. Seventy-two cannabis-smokers were matched in a 1:3 ratio to 216 non-smokers. Prior to propensity score matching, the cannabis-smokers group included fewer white patients (83.3% vs 93.4%, *p* = 0.001), younger age (54 [44, 60] vs 60 [52, 68], *p* < 0.001), more patients from distressed communities (29% vs 15%, *p* = 0.019), and higher rates of IBD (12.5% vs 5.4%, *p* = 0.012), current smokers (12.5% vs. 5.2%), COPD (16.7% vs 8.7%, *p* = 0.023), and hepatic dysfunction (4.2% vs 1.1%, *p* = 0.019). After matching, all demographics (Table 1) and operative details were similar (Fig. 1; Table 2).

**Table 1:** Demographics and comorbidities

	Cannabis smoker *n* = 72	Non-smoker *n* = 216	*p* value
Male	54% (39)	53% (114)	0.84
White race	83% (60)	82% (178)	0.86
Age, years, median (IQR)	54 (44, 60)	54 (45, 62)	0.48
BMI, kg/m^2^, median (IQR)	33 (28, 36)	33 (29, 36)	0.99
Current tobacco use	12% (9)	11% (23)	0.67
Distressed community index			
Distressed	29% (21)	19% (39)	0.25
At risk	14% (10)	21% (43)	
Mid-tier	24% (17)	22% (46)	
Comfortable	19% (14)	18% (38)	
Prosperous	14% (10)	21% (43)	
Inflammatory bowel disease	12% (9)	13% (28)	0.92
Hepatic insufficiency or liver failure	4.2% (3)	2.8% (6)	0.56
Hypertension	57% (41)	60% (129)	0.68
Diabetes	25% (18)	31% (66)	0.37
COPD	17% (12)	16% (35)	0.93
Anti-platelet medications	2.8% (2)	4.6% (10)	0.5
Anti-coagulant medications	5.6% (4)	5.1% (11)	0.88
Immunosuppressants	17% (12)	17% (36)	1
Recurrent hernia	58% (42)	60% (129)	0.83

**Table 2:** Operative details

	Cannabis smoker *n* = 72	Non-smoker *n* = 216	*p* value
Elective case	100% (72)	99.7% (216)	1
Intraoperative complication	6.9% (5)	2.8% (6)	0.11
Concomitant procedure	11.1% (8)	9.7% (21)	0.73
Hernia length, cm, median (IQR)	24 (20, 26)	23 (20, 26)	0.93
Hernia width, cm, median (IQR)	15 (12, 18)	15 (12, 20)	0.71
Mesh length, cm, median (IQR)	30 (30, 40)	30 (30, 46)	0.34
Mesh width, cm, median (IQR)	30 (30, 46)	30 (30, 50)	0.51
Fixation	57% (41)	70% (151)	0.04
Fascial closure	91.7% (66)	94% (203)	0.49
Subcutaneous drains used	27% (19)	21% (46)	0.34
Subcutaneous flaps raised	4.2% (3)	8.3% (18)	0.24

SSO rates for cannabis-smokers were 18% compared to 17% for non-smokers (*p* = 0.86). SSI (11.1% vs 9.3%, *p* = 0.65) and SSOPI (12% vs 12%, *p* = 0.92) rates were also similar. There was no difference in readmission (12.5% vs 9.3%, *p* = 0.43), or any postoperative complication (46% vs 43%, *p* = 0.63). Reoperations were more common in the cannabis-smoker group (8.3% vs 2.8%, *p* = 0.041), and were mostly related to major wound complications in the cannabis-smoker group (5/72 vs 2/216, *p* = 0.004) and postoperative bleeding in the non-smoker group (0/72 vs 3/216, *p* = 0.046). No mesh excisions occurred in this cohort (Table 3).

**Table 3:** 30 day outcomes

	Cannabis smoker *n* = 72	Non-smoker *n* = 216	*p* value
Length of stay, days, median (IQR)	5 (4, 6)	6 (4, 7)	0.21
Readmission	12.5% (9)	9.3% (20)	0.43
Reoperation	8.3% (6)	2.8% (6)	0.04
Bowel obstruction	0	1	
Major wound complication	5	2	
Postoperative bleeding	0	3	
Unrelated intra-abdominal pathology	1	0	
Mesh excision	0	0	
Any postoperative complications	46% (33)	43% (92)	0.63
SSI	11.1% (8)	9.3% (20)	0.65
SSO	18% (13)	17% (37)	0.73
SSOPI	12% (9)	12% (26)	0.92
Pulmonary embolism	1.4% (1)	1.9% (4)	0.8
Deep vein thrombosis	2.8% (2)	0.46% (1)	0.094
Myocardial infarction	0	0.93% (2)	0.41
Pneumonia	1.4% (1)	2.3% (5)	0.63
Respiratory failure	1.4% (1)	2.8% (6)	0.51

**Fig. 2:**
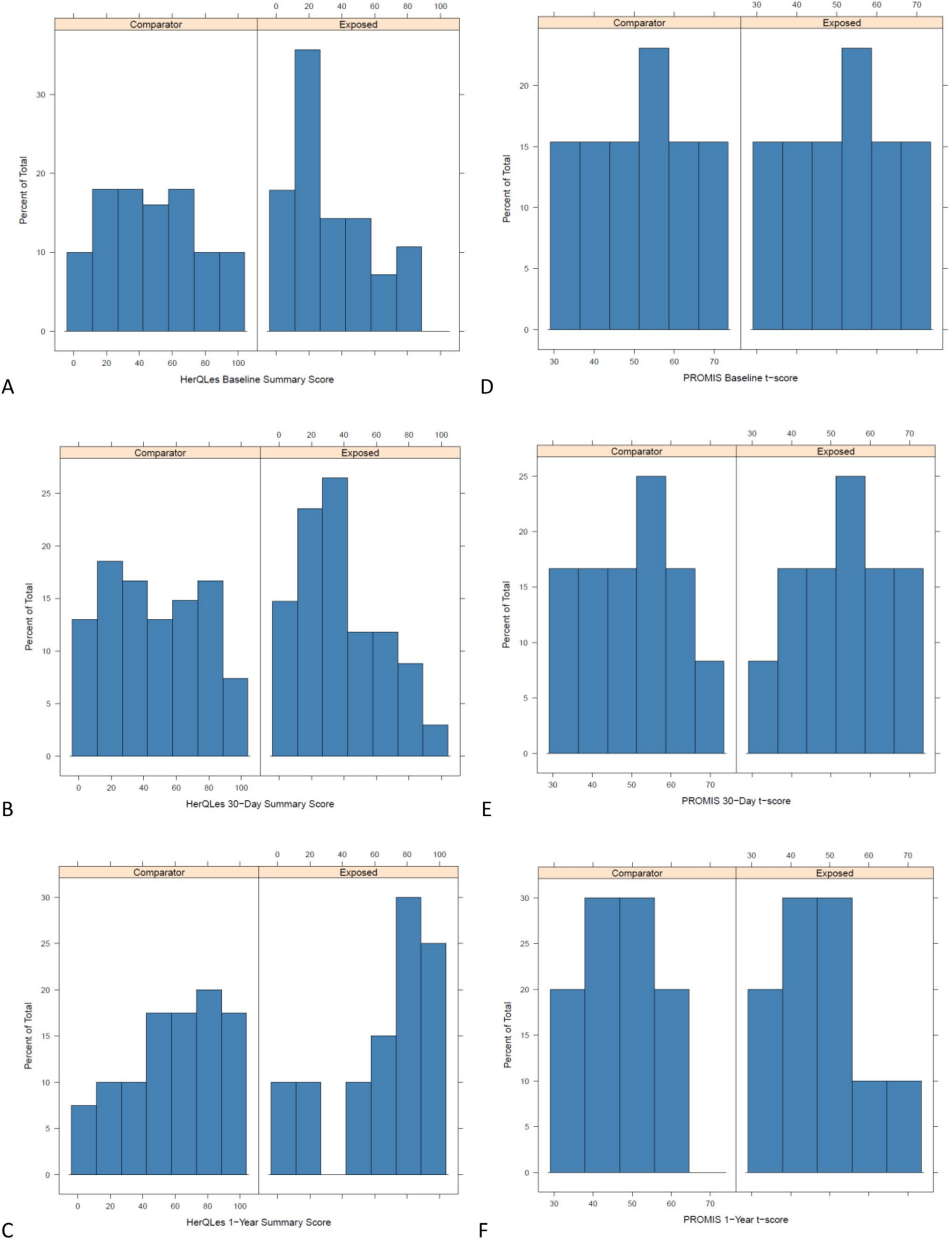
Histogram of HerQLes and PROMIS scores at baseline (**A**, **D**), 30 day follow-up (**B**, **E**), and 1 year postoperatively (**C**, **F**)

Abdominal wall-specific quality-of-life (HerQLes) scores were not significantly different at baseline for both groups (22 [11, 41] vs. 35 [14, 55], *p* = 0.06), and were worse for cannabis-smokers compared to non-smokers at 30 days (30 [12, 50] vs 38 [20, 67], *p* = 0.032), but not significantly different at 1 year postoperatively (72 [53, 90] vs 78 [57, 92], *p* = 0.39). Pain scores (PROMIS 3a-Pain Intensity) were worse for cannabis-smokers compared to non-smokers at 30 days postoperatively (52 [46, 58] vs 49 [44, 54], *p* = 0.01), but there were no differences at 6 months or 1 year postoperatively (*p* > 0.05 for all) (Table 4; Fig. 2). There were no differences in demographics for patients with any patient-reported outcomes available compared to patients with no patient-reported outcomes available (Table 5).

**Table 4:** Patient-reported outcomes

	*N*	Cannabis smoker *n* = 72	Non-smoker *n* = 216	*p* value
HerQLes score, median (IQR)				
Summary score at baseline	194	22 (11, 41)	35 (14, 55)	0.06
Summary score at 30 days	205	30 (12, 50)	38 (20, 67)	0.032
Change from baseline to 30 days	161	3.3 (− 10, 20)	6.7 (− 10, 25)	0.59
Summary score at 6 months	80	51 (19, 86)	81 (49, 92)	0.077
Change from baseline to 6 months	58	14.2 (2.5, 17.9)	40 (7.5, 55.8)	0.037
Summary score at 1 year	120	72 (53, 90)	78 (57, 92)	0.39
Change from baseline to 1 year	102	32 (11, 58)	33 (18, 58)	0.72
PROMIS score, median (IQR)				
Summary score at baseline	194	51 (44, 54)	46 (40, 54)	0.33
Summary score at 30 days	206	52 (46, 58)	49 (44, 54)	0.01
Change from baseline to 30 days	161	2.7 (− 3.1, 8.6)	0 (− 6.2, 8.6)	0.46
Summary score at 6 months	78	52 (46, 58)	40 (31, 48)	0.77
Change from baseline to 6 months	57	− 7.9 (− 9.5, − 5.6)	− 8.6 (− 13.5, 0)	0.87
Summary score at 1 year	120	38 (31, 46)	31 (31, 46)	0.65
Change from baseline to 1 year	102	− 8.2 (− 17.1, 0)	− 9.5 (− 14.5, 0)	0.96

**Table 5:** Comparison of patients with and without patient-reported outcomes

	Patient-reported outcomes available *n* = 247	Patient-reported outcomes unavailable *n* = 41	*p* value
Cannabis smoker	25% (61)	27% (11)	0.77
Male	53% (131)	54% (22)	0.94
White race	84% (207)	76% (31)	0.41
Age, years, median (IQR)	54 (45,62)	53 (44, 60)	0.54
BMI, kg/m^2^, median (IQR)	32 (29, 36)	32 (28,35)	0.36
Current tobacco use	12% (30)	5% (2)	0.17
Distressed community index			
Distressed	22% (53)	17% (7)	0.76
At risk	19% (45)	20% (8)	
Mid-tier	22% (53)	24% (10)	
Comfortable	19% (45)	20% (8)	
Prosperous	20% (47)	15% (6)	
Inflammatory bowel disease	15% (6)	7% (3)	0.25
Hepatic insufficiency or liver failure	3% (8)	2% (1)	0.79
Hypertension	60% (148)	54% (22)	0.45
Diabetes	29% (72)	29% (12)	0.99
COPD	17% (41)	15% (6)	0.75
Anti-platelet medications	3% (8)	10% (4)	0.053
Anti-coagulant medications	5% (12)	7% (3)	0.51
Immunosuppressants	17% (41)	17% (7)	0.94
Recurrent hernia	59% (145)	63% (26)	0.57

## Discussion

In this propensity score-matched study, we sought to determine if cannabis use has an association with postoperative wound morbidity or reoperations in patients undergoing clean open abdominal wall reconstruction with retromuscular placement of synthetic mesh. Although there were no major differences in overall wound complication rates, cannabis users appeared to have more severe wound complications requiring reoperation. There was a difference in patient-reported quality of life, with cannabis-smokers having worse early postoperative quality of life and pain scores which eventually normalized with longer term follow-up.

There is a gap in knowledge regarding the implications of cannabis and wound healing. In vitro models have shown that cannabidiol inhibits pro-inflammatory pathways and cannabinoid receptors have been shown to augment multiple cellular mechanism of wound healing [14, 15]. There is growing interest in the effect of cannabis on wound health with data in orthopedic literature with conflicting findings regarding outcomes following orthopedic and plastic surgeries [16–19]. Our data found similar rates of wound complications between cannabis-smokers and non-smokers, but severity of complications was worse for patients with cannabis use. More cannabis-smokers required reoperation for wound complications, though notably there were no mesh excisions in either patient group. Wound complications for patients with regular cannabis use will be important to follow to understand the clinical impact on wound healing.

Pain control following hernia repair is an important consideration for all patients but may be specifically affected by cannabis use, given known analgesic effects of cannabis and pharmacologic interactions with opioids [20–22]. Retrospective cohorts have also shown significantly higher opioid consumption and pain scores in cannabis-smokers following motor vehicle accidents, major orthopedic surgeries, IBD-related surgery, and gynecologic oncology surgery [23–28]. In chronic smokers, abstinence may also produce withdrawal which is associated with anxiety, behavioral changes, and abdominal pain, which may present diagnostic confusion postoperatively [29]. Clinical trials in surgical patients have explored cannabis as an adjunctive analgesic, without clear benefit [30–33]. Our data show a higher perception of pain in cannabis-smokers at 30 day follow-up postoperatively, which may be a reflection of a lower pain threshold or increased requirement for analgesia postoperatively. Given this observation of increased pain perception postoperatively, surgeons should counsel patients with active cannabis use regarding postoperative expectation setting for pain and analgesia management. In a similar vein, the baseline quality-of-life scores for the cannabis-smokers were surprisingly low, though not statistically significant at baseline, in this cohort and showed less improvement at 30 days and 6 months after surgery compared to non-smokers. While we are not positioned to explain causation, it is possible that the lower patient-reported quality of life is associated with increased pain perception or the higher reoperation rate. A previous prospective cohort study of elective surgical patients similarly demonstrated worse patient-reported pain and quality of life in cannabis-smokers compared to non-smokers despite similar surgical outcomes [34]. Albelo et al. similarly demonstrated worse patient-reported outcomes in multiple domains for cannabis-smokers 2 years after orthopedic surgery [35]. Supporting the idea that there is a correlation between cannabis use and quality of life prior to propensity score matching, cannabis-smokers represented significantly more distressed communities and more non-white racial groups, so it is important to acknowledge that there are likely external factors confounding quality of life for the patients with active cannabis use. Healthcare professionals have traditionally espoused negative attitudes towards patients with substance use disorders, so surgeons should be cognizant of their biases in this area and how they affect patient interactions and possibly worsen existing disparities [36].

The medical community has become increasingly aware of cannabis use in surgical patients as evidenced by a growing body of literature on the topic, and it is important for hernia surgeons to understand the implications on perioperative care and outcomes. The American Society for Regional Anesthesia (ASRA) released guidelines in 2023 on perioperative considerations for patients with cannabis use [20, 23, 37]. In addition to known pharmacologic interactions, cannabis has been shown to predispose chronic smokers to a 20% increased risk of postoperative nausea and vomiting and preoperative cannabinoid use is also associated with higher levels of postoperative pain. Cannabinoids also have significant cardiovascular effects with activating sympathetic effects in acute usage as well as increased parasympathetic tone with chronic usage that can contribute to bradycardia and hypotension and may be associated with an increased risk of postoperative myocardial infarction [38, 39]. Additionally, there may be some benefit to cannabis cessation prior to surgery, although the duration of this requires further inquiry [20, 40]. A recent cohort study of 12,422 hospitalizations after major, non-cardiac surgery found a higher incidence of composite complications and mortality in patients with cannabis use disorder (7.73% vs 6.57%) [41]. Similar large cohort NIS studies in elective spine surgery, vascular surgery, and orthopedic and bariatric surgery have also reported increased odds ratios of postoperative complications in cannabis use disorder including thromboembolic events, respiratory complications, neurologic complications, sepsis, myocardial infarction, stroke, and increased length of stay [42–45]. Our data revealed no significant differences in cardiovascular or respiratory complications in patients with known cannabis use, though our patient population was significantly different. The underlying cardiovascular and neurologic risk factors for patients undergoing vascular surgery for example are significantly different from the average profile of a patient undergoing hernia repair. Additionally, the patients included in our study did not require a diagnosis of cannabis use disorder for inclusion, which likely skews NIS data towards patients with heavy cannabis consumption. More in line with our findings, similar postoperative outcomes for cannabis-smokers and non-smokers have been demonstrated in smaller cohorts of bariatric and non-cardiac surgical patients [46–50]. The overall incidence of major medical complications in previous large database reviews was low overall, but it is important to consider the clinical significance of statistically significant p values. While a difference in composite outcomes may have led to detection of relatively rare events, the clinical significance of this finding in our patient population is unclear [41].

One of the main limitations of studying cannabis use is the risk of underreporting of substance use, which may bias the results towards the null hypothesis. At the time of this data capture, only medical cannabis use is legal in Ohio, and regardless of legal status, there is cultural stigma associated with cannabis use, both of which could disincentivize forthcoming patient-reported use. The reason for cannabis use was not captured in this study; for example the number of patients smoking for chronic pain may have influenced the rates of chronic pain and hernia-specific quality of life. Our definition of cannabis use did not distinguish frequency of use, so it is unclear from these data if outcomes might differ for routine chronic smokers versus more episodic smokers. Smoking was the only qualifying route of administration included, which restricts us from commenting on other means of ingestion, which may carry unique physiologic effects. The retrospective nature of this work presents an opportunity for selection bias and limits the follow-up; however, these data can be used for hypothesis generation and power calculation for future prospective studies. Propensity score matching adjusted for baseline differences in the cohorts but presents a risk for introducing unanticipated biases.

## Conclusion

While cannabis is a controversial issue, its accessibility and use are increasing with changes in legislation, compelling surgeons to better understand how cannabis use may affect postoperative outcomes. Active cannabis use is not prohibitive for elective open, clean ventral hernia repairs, with retromuscular synthetic mesh placement, but surgeons should take a full history of cannabis use and counsel patients about potentially higher risk for more severe wound complications. Additionally, hernia surgeons should be aware of the differences in pain perception after complex abdominal wall reconstruction in cannabis-smokers and potentially adjust expectations and perioperative management.
